# How host genetics dictates successful viral zoonosis

**DOI:** 10.1371/journal.pbio.3000217

**Published:** 2019-04-19

**Authors:** Cody J. Warren, Sara L. Sawyer

**Affiliations:** BioFrontiers Institute and Department of Molecular, Cellular, and Developmental Biology, University of Colorado Boulder, Boulder, Colorado, United States of America

## Abstract

Viruses of wild and domestic animals can infect humans in a process called zoonosis, and these events can give rise to explosive epidemics such as those caused by the HIV and Ebola viruses. While humans are constantly exposed to animal viruses, those that can successfully infect and transmit between humans are exceedingly rare. The key event in zoonosis is when an animal virus begins to replicate (one virion making many) in the first human subject. Only at this point will the animal virus first experience the selective environment of the human body, rendering possible viral adaptation and refinement for humans. In addition, appreciable viral titers in this first human may enable infection of a second, thus initiating selection for viral variants with increased capacity for spread. We assert that host genetics plays a critical role in defining which animal viruses in nature will achieve this key event of replication in a first human host. This is because animal viruses that pose the greatest risk to humans will have few (or no) genetic barriers to replicating themselves in human cells, thus requiring minimal mutations to make this jump. Only experimental virology provides a path to identifying animal viruses with the potential to replicate themselves in humans because this information will not be evident from viral sequencing data alone.

## Abstract 2

Virologists and epidemiologists view the emergence of new viral diseases in very different ways; this Essay describes two virologists' view of how animal viruses come to infect humans.

## Introduction

We are constantly exposed to animal viruses through the food that we eat, the pets that we keep, and our interactions with nature. The vast majority of viruses that enter our bodies pass harmlessly through our gastrointestinal tracts or are destroyed by our immune systems. However, on rare occasions, an animal virus encounters a human host and begins to replicate itself, executing its entire lifecycle within human cells and expanding one virion into a population of many. Replication of an animal virus within the body of this first human subject is the key moment in the zoonotic process because it renders possible two things. First, the virus will now mutate and evolve under the selective constraints of the human body for the first time, adapting and improving itself for replication in this new host. Second, high virus titers produced by viral replication mean that spread to a second human is now possible, initiating selection for variants with increased capacity to spread in the human population.

Significant effort has been put into understanding the factors that contribute to the spread of zoonotic viruses through the human population once an animal virus has begun the process described above. Factors that facilitate spread of viruses through populations can include high population density, the presence of viral vectors, and many others [1,2]. However, less investment has been made in finding the animal viruses that have the greatest potential to begin these zoonotic events in the first place. In the following essay we will argue that, while humans are constantly exposed to animal viruses, those animal viruses with real potential to replicate themselves in a human cell are exceedingly rare. We assert that host genetics plays a major role in determining which animal viruses will be able to make copies of themselves in the human body. This is because animal viruses that pose the greatest risk to humans will have few (or no) genetic barriers to replicating themselves in human cells, thus requiring minimal mutations to make this jump.

## Most animal viruses do not replicate in the human body

**Fig 1:**
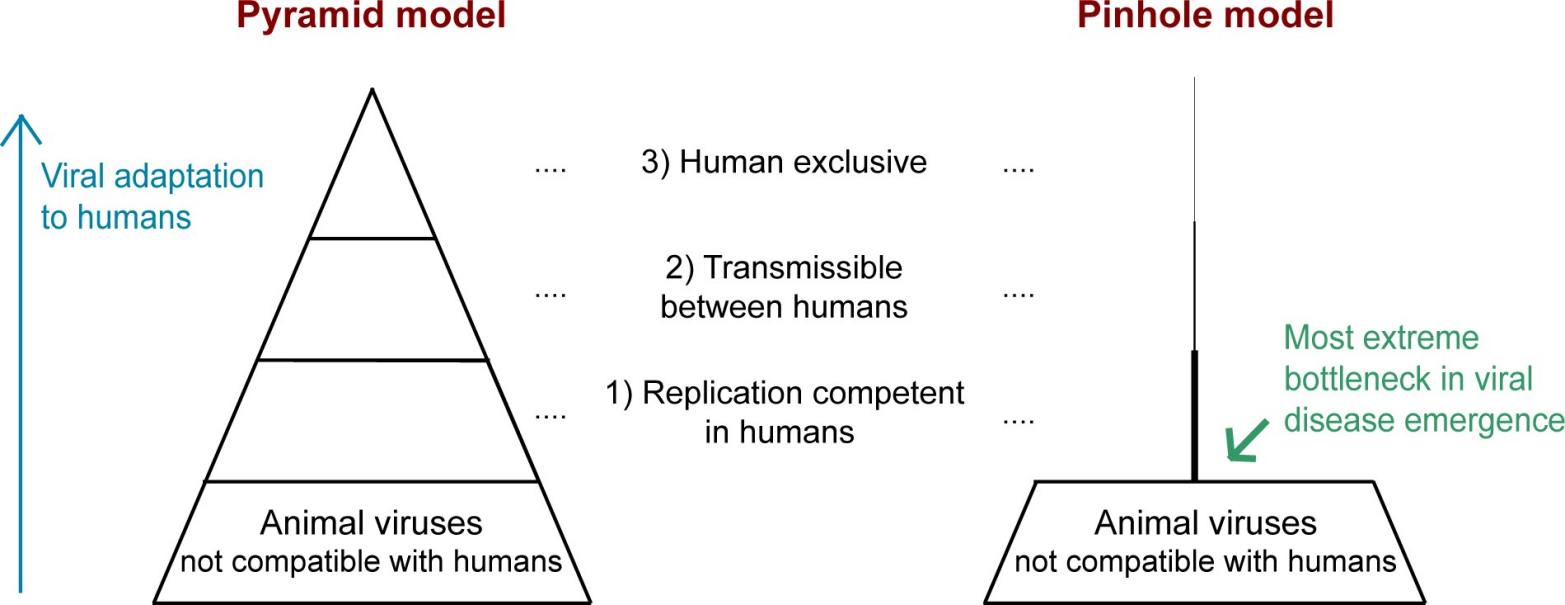
This essay focuses on the most extreme bottleneck in viral disease emergence: The replication of animal viruses in their first human host. On the left is a typical representation of the zoonosis pyramid, modified from [1,3–5]. The concept here is that animal viruses become increasingly human-adapted through a series of evolutionary steps represented from bottom to top. In our view, these steps are best described as 1) random variants arise in the animal reservoir that are capable of replicating themselves in humans, 2) variants are then selected for the ability to transmit between people, and 3) sometimes, as in the case of HIV-1 group M [12], these viruses become stably maintained in humans and are divorced from their former animal reservoir. The pyramid shape properly demonstrates that increasingly fewer viruses progress through the different stages of zoonosis but is misleading in that it doesn’t represent the scale of probabilities in these events. Instead, based on the arguments laid out in the essay, the pyramid should more correctly be depicted as a pinhole. As shown on the right, our current understanding is that <0.1% of animal viruses have any ability to replicate in humans (step 1), and then fewer still are able to meet the relevant criteria as the process continues upwards. The bottlenecks leading to the latter two steps don’t seem to be as extreme. One study of zoonotic pathogens (those already at step 1) found that 33% are transmissible between humans (step 2), and 3% spread so effectively that they become permanently sustained in humans (step 3) [10]. Herein, we discuss how experimental virology and an understanding of host–virus interactions render clear the reasons for the most extreme bottleneck in this process (green arrow).

For more than a decade, the pyramid model has been used to illustrate the stages in viral zoonosis (**Fig 1**) [1,3–5]. This model, moving upward from base to tip, highlights the steps animal viruses take to adapt to humans. While this concept is very useful, it fails to convey visually how rare zoonosis actually is. Almost all zoonotic viruses emerge from mammals or birds. There are currently 8,615 known species of mammals and 17,413 species of birds [6]. Assuming, conservatively, that there are 10 enzootic (endemic to animals) viruses per species [7], there are over 86,000 mammalian viruses and 174,000 avian viruses in nature, for a total of 260,000 animal viruses in nature. Recent estimates suggest that the actual number may be much higher, as high as 1.6 million animal viruses in nature [8,9]. In contrast, only 219 viruses have ever been documented to infect humans [4,10]. This suggests that far less than 0.1% of the animal viruses in nature have ever caused a known human infection, consistent with other recent estimates [7,11]. A modification of the pyramid model makes it visually apparent that an exceedingly small fraction of animal viruses has any path to replicating in humans (**Fig 1)**. In this pinhole model, one can more readily see why sequencing surveys of viruses in animals will do little to inform us of the ones that are dangerous to humans [9]. Based on the numbers just discussed, there appears to be a massive bottleneck between all animal viruses in nature and those that can replicate themselves in humans. Far less than 0.1% of animal viruses progress to step 1 in the process (green arrow; **Fig 1**). More research is needed, but the bottlenecks leading to the latter two steps don’t seem to be as extreme. One study of zoonotic pathogens (already at step 1) found that 33% are transmissible between humans (step 2), and 3% spread so effectively that they become permanently sustained in humans (step 3) [10]. If initial viral replication in a human is so incredibly rare and the major bottleneck in this process, then how do we identify which viruses have the potential to break through? In this essay, we discuss the reasons why the vast majority of animal viruses in nature have little or no potential to ever replicate themselves in a human host. Then, we will discuss how the exceptions can be identified.

Work in molecular virology has revealed why virus replication tends to be so specific for particular host species (**Box 1**). Viruses replicate themselves inside the cells of their hosts, and to do so, they must correctly execute tens to hundreds of protein–protein interactions. For instance, HIV is thought to interact with as many as 400 proteins in human T cells [13]. Animal viruses will replicate in human cells when they can interact with all of the useful host proteins that they need and simultaneously avoid interaction with all of the immunity proteins that will destroy them. Viruses might not interact correctly with host proteins in a new species (binding to useful proteins, avoiding immunity proteins) because of differences in the primary sequences of these proteins in the new species. Even small primary sequence differences between orthologs of a host protein can change the virus interaction surface, either directly or by changing the structure or pattern of post-translational modification, such as the addition of glycans. While an animal virus could be selected for perfecting interactions with human proteins (enhancing interaction with useful host proteins and avoiding interaction with antiviral proteins), the conditions for human adaptation will not exist until after viral replication in a human has commenced. Instead, only “alive-on-arrival” animal viruses, meaning those already able to launch replication cycles upon encountering a human, will have any opportunity to further optimize themselves for the human host. (Alive-on-arrival viruses have also been referred to as “off the shelf” [1].) Stated another way, zoonosis requires that human-compatible viral variants pre-exist in the animal reservoir, arising before these viruses have ever even experienced the selective constraints of the human body. For the vast majority of animal viruses in nature, there are just too many protein–protein interactions to master by chance in a random encounter with humans.

**Box 1. How host genetics dictates successful viral zoonosis**

Animal viruses will replicate in human cells when they can interact with all of the useful host proteins that they need and simultaneously avoid interaction with all of the immunity proteins that will destroy them. For the vast majority of animal viruses in nature, this is just too many interactions to master by chance in a random encounter with humans.

Experimental virologists know all too well how difficult it is to get viruses to infect alternate hosts. Virologists attempt “reverse zoonosis” all the time in the process of establishing animal models for human viruses, with very poor success. It is almost always impossible to get human viruses to replicate unaltered in any laboratory animal precisely because most viruses are exquisitely adapted to their natural host. HIV is a prime example. For 30 years, scientists have tried to get HIV to replicate in laboratory animals (or cells derived from laboratory animals), including numerous species of monkeys, rodents, shrews, dogs, cats, rabbits, pigs, and cows [14–18]. In this case, a major obstacle is presented by restriction factors, which are antiviral proteins of the innate immune system that are constitutively expressed, lying in wait in mammalian cells should viruses come along. The macaque monkey is the most common experimental model for HIV/AIDS research, but macaques encode at least five restriction factors (tripartite motif containing 5, alpha isoform [TRIM5α], Tetherin, SAM and HD domain containing deoxynucleoside triphosphate triphosphohydrolase 1 [SAMHD1], MX dynamin-like GTPase 2 [MX2], and apolipoprotein B mRNA-editing enzyme catalytic subunit 3G [APOBEC3G]) and one incompatible cell-surface receptor (CD4) that block HIV replication [19–26]. In addition, macaques have a massively expanded MHC class I locus compared to humans, making it extremely difficult for HIV to evade T-cell immunity and establish persistence in these animals [27–29]. Together, these factors create multiple genetic barriers to HIV in the macaque host, meaning that scientists have to alter the virus in significant ways in order to get it to replicate in these animals (**Table 1**). Likewise, many species have been explored as models for human dengue virus infection, including pigs, monkeys, and rodents [30,31]. Yet, again, dengue virus does not replicate to high titers in any of these animals [30–32]. In mice, several innate immunity proteins block dengue virus from replicating to high titers, so, in this case, scientists genetically modify mice to lack key immunity pathways [30,31] (**Table 1**). This explains one reason why mice are such a popular model organism: when no species can be found that will support the replication of a human virus, mice afford an opportunity to knock out host immunity genes in order to create an animal that will support viral replication. Additionally, mice can be modified to express human versions of proteins that support virus infection. For instance, creation of transgenic mice expressing human versions of the cellular receptors used by poliovirus and hepatitis C viruses leads to enhanced viral infection because these human viruses are not compatible with the mouse orthologs of their receptors (**Table 1**). In other cases, blocks to human viruses in animals are extreme, and it has been more practical to study related animal viruses in their natural hosts (**Table 1**). If we assume that the success of reverse zoonosis reflects the success of zoonosis, then decades of work modeling human viruses in animals would suggest that zoonosis is exceedingly difficult to achieve.

**Table 1:** Animal models of human viruses as examples of attempted “reverse zoonoses.”

Human Virus	Model	Genetic Blocks in Animal Model	Steps Taken to Overcome Blocks in Animal Models	References
HIV	Macaque	Multiple barriers including restriction factors, CD4 receptor, and an expanded MHC class I locus	**Use monkey virus instead or modify the HIV genome** SIVmac or SHIVs	[33,34]
Dengue virus	Mouse	Innate immunity	**Modify mice** Create transgenic mice that lack STAT2 or are knocked out for IFNα, -β, -γ	[35,36]
Poliovirus	Mouse	Cellular receptor PVR	**Modify mice** Create transgenic mice that express human PVR receptor	[37]
Hepatitis C virus	Mouse	Cellular receptors CD81, OCLN; innate immunity	**Modify mice** Create transgenic mice that express human CD81, OCLN receptors, and lack STAT1	[38]
Epstein–Barr virus/Kaposi sarcoma herpesvirus	Mouse	Highly species specific in pathogenicity and disease	**Study mouse virus instead** MHV-68	[39]
Human papillomavirus	Mouse	Highly species specific in pathogenicity and disease	**Study mouse virus instead** MmuPV1	[40]

**Abbreviations**: IFN, interferon; MHV-68, murine gammaherpesvirus-68; MmuPV1, *Mus musculus* papillomavirus 1; OCLN, occludin; PVR, PVR cell adhesion molecule; SHIV, SIVmac/HIV chimeric virus; SIVmac, simian immunodeficiency virus of macaques; STAT1, signal transducer and activator of transcription 1; STAT2, signal transducer and activator of transcription 2; MHC, major histocompatibility complex.

Alternately, perhaps humans have historically been infected by very few animal viruses (<0.1% of the total in nature) simply because the opportunity for animal viruses to infect humans is very limited. Certainly, an animal virus that never comes into contact with humans would have no path for infecting them. If opportunity is key to understanding the main bottleneck in zoonosis (green arrow, pinhole model, **Fig 1**), then we would expect that zoonosis would occur more commonly from the animals that we domesticate, and indeed this does appear to be true [41]. Yet our relationship with animals goes well beyond domestication. In a world of over 7 billion people, there are a staggering number of collisions occurring between people and animals every day. Further, human overcrowding has increased the chances that zoonotic viruses will circulate in human populations long enough to be detected. Yet, still, only 219 viruses have ever been identified in human infections [4,10]. Rather than opportunity being limiting, it’s more plausible that we are actively protected from the vast majority of these collisions with animal viruses. In the following section, we discuss the remarkable ways that the human body defends itself against animal viruses. Notably, what protects us from animal viruses is often different than what protects us from human viruses.

## What protects humans from animal viruses?

The adaptive immune system (protection mediated by B and T lymphocytes) harbors memory of past infections and ensures a robust and specific response upon secondary exposure. Adaptive immunity is likely important for limiting human disease caused by animal virus infection. However, infection with a new animal virus represents a first exposure, and the adaptive immune system will take several days to activate and mediate an effective response. By the time the adaptive immune response kicks in, a swarm of viral variants will exist in the newly infected human, and this swarm may contain variants with improved intracellular replication or even immune escape. Certainly, more work is needed in understanding the role of adaptive immunity in overcoming zoonotic infections. Since this essay especially focuses on the key event of initial viral replication cycles in a first human host, we can at least speculate that adaptive immunity is unlikely to be relevant here. Only after high virus titers have been reached will an adaptive immune response be mounted.

Nonspecific immune barriers, including skin, mucosa, and chemical defenses (for example, stomach acid), play a key role in blocking animal viruses at portals of entry into the human body. However, even if these barriers are penetrated and an animal virus gains access to human cells of its target tissue type, productive replication is still far from guaranteed. To launch even a first round of viral replication, animal viruses will need to avoid interaction with a suite of constitutively expressed human innate immunity proteins present inside of human cells, namely restriction factors and pathogen sensors that can activate the interferon response. If the animal virus is successfully recognized by any of these human proteins, this will potently block viral replication and will limit even initial rounds of animal virus replication. For example, several human restriction factors are efficient at blocking replication of primate-derived simian immunodeficiency viruses (SIVs), and this was important in the emergence of HIV in humans. SIV from chimpanzees (SIVcpz) adapted to overcome the human restriction factors APOBEC3H and Tetherin, giving rise to pandemic HIV-1 group M (**Box 2**). The result is that HIV is now insensitive to these factors in humans [42,43]. As such, restriction factors may provide immediate protection against animal viruses but tend to be less useful once these viruses have adapted to humans and evolved to evade them. For this reason, restriction factors and other components of innate immunity seem to be highly relevant to protecting us from zoonotic viruses and less relevant to protecting us from human viruses [23,44,45]. The complex array of restriction factors and innate immune sensors encoded in the human genome means that most animal viruses would need to escape multiple immunity proteins simultaneously. For this reason, animal virus escape from human innate immunity is probably quite rare.

**Box 2. Lessons in zoonosis from the HIV pandemic**

**HIV adapted to humans as it emerged from its reservoir in monkeys and apes.** HIV-1 group M, the virus responsible for the HIV/AIDS global pandemic, derives from a reservoir of SIVs found in African monkeys. Monkey SIV first entered African apes via infection of chimpanzees [46]. This transmission resulted in a new virus of chimpanzees called SIVcpz. SIVcpz was then transmitted to humans, giving rise to HIV-1 group M [47]. Given the enormity of the pandemic that ensued, these events have been intensely scrutinized by the HIV research community, and this topic has been recently reviewed [48].

We know a lot about how the virus adapted as it moved from monkeys to chimpanzees and then from chimpanzees to humans. For instance, in the adaptation of monkey SIV to chimpanzees, the APOBEC3 family of host restriction factors was key. The SIV/HIV protein Vif neutralizes APOBEC3 proteins in host cells. SIV from monkeys could not neutralize chimpanzee APOBEC3D and APOBEC3G proteins, and so the vial *vif* gene had to adapt before or during this transmission event [49]. A second host protein, RanBP2, also drove viral adaptation during this transmission event. RanBP2 is part of the nuclear pore complex, and the transmission of SIV from monkeys to chimpanzees required new mutations in the viral capsid protein that enabled the virus to interact with the chimpanzee version of RanBP2 [50].

Then, during the transmission of SIVcpz to humans, protein sequence differences between chimpanzee and human Tetherin, another host restriction factor, also required viral adaptation. The virus adapted its *vpu* gene, which encodes an antagonist of human Tetherin [51,52]. Similarly, human APOBEC3H drove adaptive change in the viral *vif* gene encoding the antagonist for APOBEC3 proteins [53]. We don’t know whether these *vif* adaptations were required before the first human infection or afterwards as the virus refined itself for spread in the human population. This is because many people, including many Africans, encode APOBEC3H alleles that do not express high levels of protein [43]. It’s possible that these individuals provided a lower adaptive barrier for SIVcpz, giving the virus a foothold in the human population and a chance for the resulting HIV to acquire changes in *vif* that subsequently enabled it to infect more humans [43,53]. In the transmission of SIVcpz to humans, there was a third key adaptive event in SIV matrix protein, although the host factor driving this adaptation remains unknown [54].

In addition to avoiding adaptive and innate immune defenses, zoonotic animal viruses must also successfully interact with and recruit tens to hundreds of host proteins that the virus needs in order to replicate itself in human cells (for example, nuclear import factors, transcription factors, etc.). If the human version of even one of these essential host factors cannot be recruited by an animal virus because of differences in protein sequence between the human and animal version of that protein, then this, too, can impede viral replication. This is probably the main way that immunity to animal viruses differs from immunity to human viruses. Something as simple as a human transcription factor, mismatched to the animal virus attempting to use it, can be as potent in blocking viral replication as an immunity protein would be. For instance, the host transcriptional activator acidic nuclear phosphoprotein 32 family member A (ANP32A) is a key cofactor required for influenza virus replication [55,56]. Avian influenza viruses require a mutation in their PB2 polymerase subunit protein in order to render the avian virus polymerase complex compatible with the human version of ANP32A (**Table 2**; [55]). Without this mutation, this human transcriptional activator provides a potent block to avian influenza virus replication in humans. As another example, RanBP2 is a component of the nuclear pore complex, and HIV engages with Ran binding protein 2 (RanBP2) to facilitate nuclear entry [57–60]. We have shown that RanBP2 is nonequivalent in sequence and interaction specificities between monkeys, chimpanzees, and gorillas and that this nonequivalence drove the evolution of SIVs as they transmitted into and between ape species, setting the stage for zoonosis to humans and the emergence of HIV (**Box 2**). Because the viral capsid protein interacts with RanBP2, we found that the viral adaptive mutations acquired during transmission to and between different ape species mapped to capsid [50]. Table 2 shows a collection of human proteins that have been shown to block the replication of animal viruses in human cells. While some of them are restriction factors, others are core housekeeping proteins, such as cell-surface receptors, that are not typically thought of as canonical members of the immune system. Even if these receptors do function in immune signaling, that activity is typically not what is blocking replication of animal viruses. Rather, it’s the inability of animal viruses to physically engage these receptors that impedes virus entry into cells and ultimately viral replication. The final outcome is a highly effective defense against these animal viruses, in some cases conveyed by relatively mundane housekeeping proteins of the host.

**Table 2:** Some of the human proteins known to block replication of animal viruses.

Animal Virus	Barrier to Replication in Human Cells	Viral Mutations that Overcome Barrier^a^
Avian influenza viruses	α-2,6–linked sialic acid receptor	Mutations in receptor binding site of viral HA One or a combination of mutations in HA is required; several different combinations have been observed [61–63]
	ANP32A host cofactor for the viral polymerase	Mutations in viral PB2 PB2-E627K, others observed as well [63–65]
SIVcpz	APOBEC3H restriction factor	Mutations in viral Vif [48,53]
	Tetherin restriction factor	Mutations in viral Vpu [48,51,52]
Bat coronaviruses	ACE2 receptor	Mutations in viral surface glycoprotein Spike S1 [66,67]
Rodent New World arenaviruses	TfR1 receptor	Mutations in viral surface glycoprotein [68]
Ebola virus (presumably from bats)	NPC1^b^ receptor	Mutation in viral surface glycoprotein [69–71]^b^
Feline leukemia virus	APOBEC3 restriction factor	Not yet zoonotic (escape path not yet known) [72]

^a^Mutations that each virus gained to overcome these barriers in humans.

^b^This Ebola glycoprotein mutation may only be necessary to optimize human transmission after zoonosis since Ebola virus acquired this mutation during the course of a major human epidemic. **Abbreviations**: ACE2, angiotensin I converting enzyme 2; ANP32A, acidic nuclear phosphoprotein 32 family member A; APOBEC3, apolipoprotein B mRNA editing enzyme catalytic subunit 3; HA, hemagglutinin; NPC1, NPC intracellular cholesterol transporter 1; SIV, simian immunodeficiency virus; SIVcpz, SIV from chimpanzees; TfR1, transferrin receptor 1; viral infectivity factor encoded by HIV/SIV.

## How do we identify the animal viruses best poised to replicate themselves in humans?

The key to identifying the animal viruses that are most likely to harm humans boils down to understanding “thick” versus “thin” barriers to zoonosis. We must identify the human proteins (restriction factors, receptors, other cellular proteins) that are currently protecting us from each class of animal virus with high zoonotic potential. Such blocks may include restriction factors like APOBEC3H or Tetherin, or incompatible host proteins like ANP32A or RanBP2, all of which were discussed above. Based on this, we can then determine for which animal viruses our resistances are few (a thin barrier to zoonosis) versus many (a thick barrier to zoonosis). Based on historical zoonoses (a partial list is shown in **Table 3**), it is straightforward to identify the predominant virus families on which these efforts should be focused. Identifying genetic blocks to animal virus replication in humans will require significant laboratory experimentation. Furthermore, the human blocks will be unique for each animal virus of interest. While it may sound laborious, virologists are already well on their way to identifying genetic barriers that protect humans from key families of animal viruses (**Table 2**).

**Table 3:** Examples of animal viruses that are known to have infected humans.

	Zoonotic Viruses Animal Viruses that Are Known to Have Infected Humans^a^		Divorced from Animal Reservoir But Known to Derive from Animals^b^
**Arenaviridae** zoonotic arenaviruses typically associated with rodents	Chapare virus Dandenong virus Guanarito virus Junin virus Lassa virus Lujo virus	Lymphocytic choriomeningitis virus Machupo virus Sabia virus	
**Filoviridae** animal associations poorly understood	Bundibugyo virus Marburg virus Reston virus	Sudan virus Tai Forest virus Ebola virus	
**Hantaviridae** zoonotic hantaviruses typically associated with rodents	Amur virus Andes virus Araraquara virus Bayou virus Bermejo virus Black Creek Canal virus Castelo Dos Sonhos virus Choclo virus Dobrava-Belgrade virus Hantaan virus Juquitiba virus Laguna Negra virus	Lechiguanas virus Maciel virus Monongahela virus Muleshoe virus New York virus Oran virus Puumala virus Saaremaa virus Seoul virus Sin Nombre virus Thailand hantavirus Tula virus	
**Coronaviridae** zoonotic coronaviruses typically associated with bats	MERS coronavirus	SARS coronavirus	Coronavirus 229E Coronavirus OC43 Coronavirus NL63 Coronavirus HKU1
**Paramyxoviridae** zoonotic paramyxoviruses typically associated with bats	Avian paramyxovirus-1 Hendra virus Menangle virus	Nipah virus Tioman virus	Measles virus Mumps virus Respiratory syncytial virus Parainfluenza viruses Human metapneumovirus
**Flaviviridae** zoonotic flaviviruses typically vectored by mosquitos or ticks	Alkhumra virus Banzi virus Bussuquara virus Edge Hill virus Ilheus virus Japanese encephalitis virus Kokobera virus Koutango virus Kyasanur forest disease virus Langat virus Louping-ill virus Murray Valley encephalitis virus	Omsk hemorrhagic fever virus Powassan virus Rocio virus Sepik virus Spondweni virus St. Louis encephalitis virus Sylvatic dengue virus Tick-borne encephalitis virus Usutu virus Wesselsbron virus West Nile virus Yellow fever virus Zika virus	Dengue virus 1–4 Hepatitis C virus
**Orthomyxoviridae** zoonotic orthomyxoviruses typically associated with birds	Avian influenza A viruses (multiple) Dhori virus	Quaranfil virus Thogoto virus	Influenza A, B, C
**Retroviridae** zoonotic retroviruses typically associated with primates	SIVcpz SIVgor	Simian foamy virus Simian T-lymphotropic viruses	HIV-1 HIV-2 HTLVs

^a^Sometimes, these viruses have caused only a single known human infection.

^b^Viruses in this column have become divorced from the animal reservoir, meaning that humans alone (or humans and an arthropod vector) are now sustaining the virus over time. **Abbreviations**: HTLV, human T-lymphotropic virus; MERS, Middle East respiratory syndrome; SARS, severe acute respiratory syndrome; SIV, simian immunodeficiency virus; SIVcpz, SIV of chimpanzees; SIVgor, SIV of gorillas.

Dangerous animal viruses are those that require zero or only a few mutations in order to begin replicating themselves in human cells. They are dangerous because these mutational combinations might arise in the animal reservoir or in an intermediate host species, creating alive-on-arrival viral variants that can begin replicating upon encountering a human host. It is critical to note that these mutational combinations must arise without the advantage of being selected for within the human body, because selection can only occur after replication in humans has begun. Further, all necessary mutations for replication in humans would need to appear together in the same viral variant arising in nature. With these criteria in mind, it is easy to see why animal viruses are highly unlikely to randomly acquire compatibility with humans when there are many different host blocks to overcome. This is, in fact, the basis of successful treatment of HIV infections with multidrug cocktails; while HIV can easily mutate to escape one drug, no single viral genome acquires the multiple drug escape mutations required for viral replication in a multiple-drug–treated host. Alarmingly, some of the H5N1 influenza viruses that are currently circulating in the avian reservoir already harbor PB2 mutations that would make them compatible with the human ANP32A [64,73]. Likewise, it is known that bat coronaviruses require mutations in their surface glycoprotein (spike) in order to use the human ortholog of their receptor, ACE2 (**Table 2**). But when coronoviruses are sampled from wild bats, a small number of them already encode spike variants compatible with human ACE2 [74,75]. The critical question is, how many additional obstacles (if any) do these viruses need to overcome to replicate themselves in human cells?

Within the vast constellation of host–virus interactions described herein, how do we find the human proteins that block replication of a particular animal virus of concern? Here, we have one critical advantage: only proteins that bind viruses differentially between the old (animal) and new (human) hosts are relevant to zoonosis. A certain host transcription factor may be critically important to the replication of a virus, but if the virus can engage that transcription factor equally in its animal host and in humans, then it is not relevant to zoonosis. Indeed, the vast majority of mammalian proteins are highly conserved in sequence because they perform important functions that constrain their evolution. Viruses are expected to interact with animal and human versions of most host proteins equivalently. Thus, the vast majority of critical host–virus interactions will not be relevant to zoonosis.

However, in every host genome, some genes get trapped into a different mode of evolution in which they are selected for continual sequence change at the protein level. This occurs in genes that are engaged with ever-changing forces in the external world, with classic examples being genes that help us to discriminate new food sources, evade new predators, or resist new pathogens [76]. These types of dynamic external forces can place intense selective pressure on certain classes of host genes, such as odorant receptors and innate immunity genes, to encode proteins with ever-new interaction specificities. Viral restriction factors and innate immune sensors are often evolving under this type of “positive” natural selection [52,77–84]. Our lab and others have shown that rapidly evolving host proteins can be potent blocks to virus host switching, and this is due to nonequivalency in virus interaction surfaces from one species to the next. Further, these proteins can usually be identified bioinformatically by a special evolutionary signature contained in their gene sequence (dN/dS > 1, indicating that natural selection is acting in favor of nonsynonymous substitutions, dN, relative to the baseline rate of synonymous substitutions, dS). Identifying this dN/dS > 1 signature in mammalian genes, in particular those important for virus biology, has proven itself to be a powerful shortcut in identifying host proteins that interact differently with viruses in one possible animal host versus another [32,44,45,50,78,81,82,85–97]. Predictions based off of the identification of host genes with dN/dS > 1 must still be tested in the lab, but this combined bioinformatic and experimental approach can ultimately allow us to identify dangerous animal viruses for which human blocks are few rather than many. Most of the human proteins known to block the replication of animal viruses (partial list in **Table 2**) are evolving under positive selection [52,93,95,96,98] or exhibit other types of significant sequence divergence between the reservoir animal and human orthologs. (For instance, most avian versions of ANP32A have 33-amino–acid insertion compared to mammalian versions [55]). The beauty of this, therefore, is that candidate human proteins that are protecting us from animal viruses can be identified bioinformatically, usually by a signature of dN/dS > 1 in their gene sequence.

**Fig 2:**
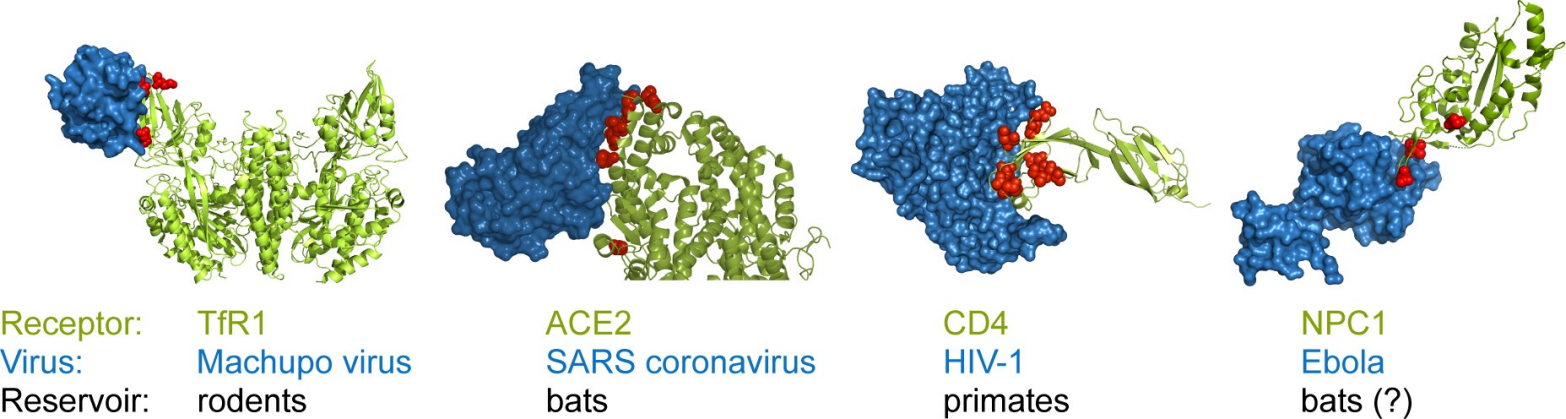
Receptors used by viruses are under positive selection at the host–virus interaction interface. The cocrystal structures are shown for four human cellular receptors (green) in complex with the surface glycoproteins (blue) of four viruses that use these receptors to enter cells. In each case, we analyzed the evolution of the receptor gene throughout the speciation of the animals that have served as long-term reservoirs for each of these viruses (rodents, bats, and primates in these four cases). In those analyses, amino-acid positions in the receptors that are evolving under positive selection (dN/dS > 1; red spheres) were identified that mapped directly to the interaction interface with the indicated viruses [93–96]. These residue positions are rapidly evolving, which means that each species within these animal groups tends to encode unique amino acids at these positions. This explains why viruses often have to accumulate mutations in their surface glycoprotein that allow them to use the version of their receptor in a new species. Cocrystals shown are TfR1 (PBD: 3KAS [103]), ACE2 (PDB: 2AJF [104]), CD4 (PDB: 1RZJ [105]), and NPC1 (PDB: 5F1B [106]). The rendering of the CD4 cocrystal is from [92]. ACE2, angiotensin I converting enzyme 2; dN, rate of nonsynonymous substitution; dS, rate of synonymous substitution; NPC1, NPC intracellular cholesterol transporter 1; PDB, Protein Data Bank; SARS, severe acute respiratory syndrome; TfR1, transferrin receptor 1.

Viral receptors serve as an excellent demonstration of this approach. One of the predominant themes that has arisen in the study of zoonosis is that cell-surface receptors are commonly protecting humans from infection by animal viruses. To identify and enter their target cells, viruses interact with receptors (usually, proteins) expressed on the surface of host cells. We and others have observed that many host receptors for virus entry have evolved under intense selective pressure to modify the protein sequence on their virus-binding surface [86,93,95–97,99]. In these cases, most of the protein sequence is conserved, yet some residues at the virus interaction interface are rapidly evolving (dN/dS > 1; red spheres in **Fig 2**). Because of sequence divergence on the surface of cellular receptors, animal viruses often require compensatory mutations in their surface proteins before they can utilize the ortholog of their entry receptor in a new host species (for an interesting recent example, see [100]). Such adaptations have been well documented for bat coronaviruses, which must adapt to use the human ortholog of their receptor, ACE2 [101,102], and rodent arenaviruses, which must adapt to the human ortholog of their receptor, transferrin receptor (TfR1) [68] (**Table 2**). Similarly, Ebola virus, presumably transmitting from bats, has also mutated to refine its interaction with the human ortholog of its NPC1 receptor after it entered the human population (**Box 3**) [69–71]. But why have receptors experienced such strong natural selection at these interfaces? Once inside a human cell, viruses potentially have access to everything they need to replicate. Because even a poorly replicating virus has the ability to evolve and adapt, keeping foreign viruses completely out of cells might be one of the most important defense strategies of all. This might explain why receptors have experienced such intense selective pressure.

**Box 3. Lessons in zoonosis from the 2013–2016 Ebola epidemic**

**Kids and bats meet in a hollowed-out tree.** In the 2013–2016 Ebola virus epidemic, the index case was a 2-year-old boy in the village of Meliandou, Guinea, who died on December 28, 2013 [109]. Epidemiological investigation revealed that this toddler and other children in the village liked to play in an old, hollowed-out tree, one that was also the home of a colony of Angolan free-tailed bats (*Mops condylurus*; [109]). Although bats have never been definitively shown to be a reservoir for Ebola, this particular bat species has been found to harbor antibodies to Ebola virus in nature [110] and also to survive experimental infections with Ebola virus [111]. The tree may have put this boy in contact with infected bat feces or even with an Ebola-infected bat because the villagers reported that the children would regularly catch and play with the bats in this tree. Interestingly, a new ebolavirus, Bombali virus, was recently detected in this same bat species [112].

**Did a viral variant arise in this tree that was capable of replicating in humans?** Villagers report that both kids and bats had frequented this tree for many years. So, what exactly happened in December of 2013, when the toddler became infected with Ebola virus? Perhaps the bats in this tree had, themselves, only become infected recently or were shedding higher-than-normal levels of virus for some reason. But there is another possibility. Viruses exist as swarms of related genotypes in nature. These swarms are the pools from which variants with unique abilities can arise. Perhaps one of these bats was harboring a unique viral variant that was poised for success in a human host. We will never know for sure if the bats in this tree harbored Ebola virus or if the virus that they harbored was special in its sequence because the tree was burned before it could be properly investigated [109].

**Was Ebola virus perfecting itself for humans as the epidemic progressed?** About 3 months after the first toddler died, sequence data from the epidemic revealed the appearance of a new mutation in Ebola virus genomes recovered from infected patients. This mutation caused a single amino-acid change, A82V, in the surface glycoprotein of Ebola virus, which increased viral fusion with human cells via the endosomal receptor NPC1 [69–71]. Presumably this virus, which had just emerged from nature, needed to refine interactions with the human ortholog of its receptor, NPC1. Reciprocally, Ebola virus isolated from human patients is incompatible with NPC1 in at least one bat species [93]. Therefore, adaptation to NPC1 seems to be required during the transmission of Ebola viruses between hosts in multiple directions.

In summary, receptors and innate immunity proteins are prominent examples of proteins that block the replication of animal viruses in humans. But any cellular protein important to virus biology that has diverged significantly in protein sequence between the animal host and humans has the potential to act as a barrier to zoonosis. Other, unexpected types of host proteins have been identified that are important for viruses but that don’t translate from one host species to the next, such as DNA repair proteins [91], nuclear pore proteins [50,107], RNA nucleases [90], and proteins involved with translation and post-translational modification [89,108].

## Extrinsic factors fuel the spread of zoonotic viruses, but only after replication in humans has commenced

Once an animal virus has achieved measurable titers in its first human host, it is possible that a second human will become infected through contact with the first. Ultimately, this virus might go on to infect only a small handful of people or millions of people at the other extreme. Once epidemics have begun, the spread of viral pathogens through populations is an elongated and complex process, with many factors at play. At this point, the virus population will experience selection for variants with increased capacity to spread through the human population. As Don Ganem put it, “What evolution is operating on is not disease, disease is incidental. It operates on spread” [113]. How well a virus spreads in a new population (quantified by the reproductive number, R_0_) depends on two important factors: 1) how well the virus is adapted for transmission in the new host species and 2) the external (epidemiological and ecological) factors that facilitate spread in the new host. For instance, a less pathogenic influenza virus variant that causes lower levels of inflammation and cytopathic effects in the respiratory tract may not be transmitted well. This is because inflammation and cytopathology contribute to increased airway congestion, resulting in coughing fits, sneezing, and increased nasal secretions, which serve as major routes for influenza virus transmission. However, factors external to the virus are also integral to transmission. If a poorly spreading virus, like the influenza variant just mentioned, emerged in a densely populated megacity, then it might be capable of continued spread and further refinement in humans. On the other hand, if an influenza virus variant arises with characteristics that make it excellent at spreading in humans, but this event occurs in a remote part of the world with low population density, then the virus may falter very quickly.

**Fig 3:**
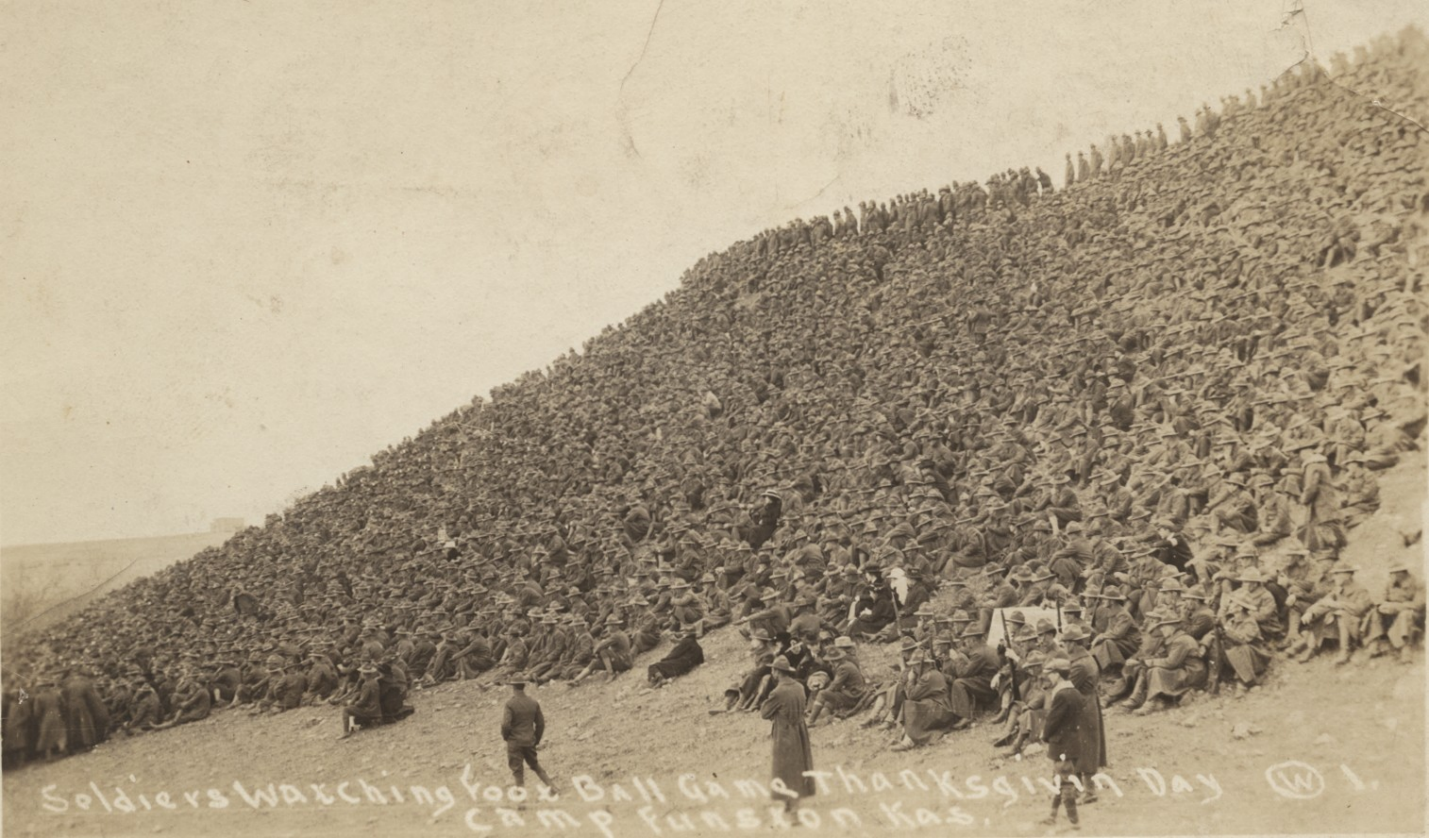
The emergence of the 1918 flu during World War I. This photo shows soldiers at Camp Funston in Fort Riley, Kansas, in late November of 1918. This camp is where some of the very first cases of the 1918 flu were reported, earlier in the year of 1918, and then again just around the time that this photo was taken [118]. Based on this, some of the soldiers shown are very likely infected with the 1918 influenza virus at the time the photo was taken. The soldiers are watching a Thanksgiving football game, and it is interesting to speculate that this dense human crowd may have set the 1918 flu, a respiratory virus, onto its disastrous course. Photo reproduced with permission from the National World War I Museum and Memorial, Kansas City, Missouri, U.S.A.

Unique human situations and behaviors are often factors in defining the scale of epidemics. The strong human desire to care for sick family members has promoted the spread of Ebola virus whenever it strikes, and it has been heartbreakingly stated that “one of the tragedies of Ebola is that it spreads through love” [114]. The 1918 influenza virus emerged during World War I and spread well between soldiers living at high density on military bases (**Fig 3**). These examples show how parameters extrinsic to the virus itself are critical to the spread of zoonotic viruses in humans [115]. If we do begin to identify the relatively rare animal viruses with thin genetic barriers to replication in human cells, then we may be able to use global data on key epidemiological and ecological parameters to understand where conditions exist that will promote rapid spread once animal virus replication in an initial human host has occurred [116,117].

In summary, dangerous animal viruses are those that require few mutations in order to begin replicating themselves in human cells. These viruses are dangerous because the required combination of mutations might randomly arise in the natural reservoir. To identify these dangerous viruses, we must identify the human proteins (restriction factors, receptors, other cellular proteins) that are currently protecting us from each class of animal virus with high zoonotic potential and determine whether those are few or many. Dangerous viruses will be separated from us by only one or a few host blocks. Identifying human blocks to animal virus replication will require significant work, including the integration of viral surveillance with virologic experimentation in the lab. These efforts are worth it, not only because disease emergence is an important public health topic, but because zoonosis provides a unique example of evolution in action that teaches us about biology more generally.

## Abbreviations

ACE2: angiotensin I converting enzyme 2
ANP32A: acidic nuclear phosphoprotein 32 family member A
APOBEC3: apolipoprotein B mRNA editing enzyme catalytic subunit 3
dN: rate of nonsynonymous substitution
dS: rate of synonymous substitution
HA: hemagglutinin
HTLV: human T-lymphotropic virus
MERS: Middle East respiratory syndrome
MHV-68: murine gammaherpesvirus
MX2: MX dynamin like GTPase 2
NPC1: NPC intracellular cholesterol transporter 1
OCLN: occludin
PB2: influenza virus polymerase PB2
PDB: Protein Data Bank
PVR: PVR cell adhesion molecule
RanBP2: RAN binding protein 2
SAMHD1: SAM and HD domain containing deoxynucleoside triphosphate triphosphohydrolase 1
SARS: severe acute respiratory syndrome
SHIV: SIVmac/HIV chimeric virus
SIV: simian immunodeficiency virus
SIVcpz: simian immunodeficiency virus of chimpanzees
SIVgor: simian immunodeficiency virus of gorillas
SIVmac: simian immunodeficiency virus of macaques
STAT1: signal transducer and activator of transcription 1
STAT2: signal transducer and activator of transcription 2
TfR1: transferrin receptor 1
TRIM5α: tripartite motif containing 5, alpha isoform
